# Effects of Renal Denervation Documented in the Austrian National Multicentre Renal Denervation Registry

**DOI:** 10.1371/journal.pone.0161250

**Published:** 2016-08-16

**Authors:** David Zweiker, Thomas Lambert, Clemens Steinwender, Thomas Weber, Markus Suppan, Helmut Brussee, Christian Koppelstätter, Julia Kerschbaum, Bruno Watschinger, Katharina Hohenstein-Scheibenecker, Roman Reindl-Schwaighofer, Thomas Sturmberger, Claudia Kindslehner, Thomas Werner Weiss, Miklos Rohla, Peter Gruener, Petra Maister, Johann Auer, Cornelia Dechant, Josef Sykora, Christoph Krismer, Stefan Glaser, Robert Zweiker

**Affiliations:** 1 Division of Cardiology, Department of Internal Medicine, Medical University Graz, Graz, Austria; 2 Department of Internal Medicine I, Kepler University Hospital, Linz, Austria; 3 Department of Internal Medicine II, Klinikum Wels-Grieskirchen, Wels, Austria; 4 Department of Internal Medicine III, Medizinische Universität Innsbruck, Innsbruck, Austria; 5 Department of Internal Medicine IV, Medizinische Universität Innsbruck, Innsbruck, Austria; 6 Division of Nephrology, Department of Internal Medicine III, Medical University Vienna, Vienna, Austria; 7 Department of Internal Medicine II, Krankenhaus der Elisabethinen Linz, Linz, Austria; 8 Department of Internal Medicine, Landeskrankenhaus Waidhofen an der Ybbs, Waidhofen/Ybbs, Austria; 9 Third Department of Internal Medicine, Wilhelminenspital and Medical Faculty, Sigmund Freud University, Vienna, Austria; 10 Medical Faculty, Sigmund Freud University, Vienna, Austria; 11 Department of Internal Medicine II, Paracelsus Medical University Salzburg, Salzburg, Austria; 12 Department of Internal Medicine I, Krankenhaus St. Josef Braunau, Braunau, Austria; 13 Fifth Medical Department, Kaiser-Franz-Josef-Spital, Vienna, Austria; 14 Department of Internal Medicine, Privatklinik Mariahilf, Klagenfurt, Austria; 15 Department of Internal Medicine, Krankenhaus St. Vinzenz, Zams, Austria; 16 Department of Internal Medicine II, Landesklinikum Wiener Neustadt, Wiener Neustadt, Austria; Shanghai Institute of Hypertension, CHINA

## Abstract

Renal denervation (RDN) is a new procedure for treatment-resistant hypertensive patients. In order to monitor all procedures undergone in Austria, the Austrian Society of Hypertension established the investigator-initiated Austrian Transcatheter Renal Denervation (TREND) Registry. From April 2011 to September 2014, 407 procedures in 14 Austrian centres were recorded. At baseline, office and mean 24-h ambulatory blood pressure (ABP) were 171/94 and 151/89 mmHg, respectively, and patients were taking a median of 4 antihypertensive medications. Mean 24-h ABP changes after 2–6 weeks, 3, 6 and 12 months were -11/-6, -8/-4, -8/-5 and -10/-6 mmHg (p<0.05 at all measurements), respectively. The periprocedural complication rate was 2.5%. Incidence of long-term complications during follow-up (median 1 year) was 0.5%. Office BP and ABP responses showed only a weak correlation (Pearson coefficient 0.303). Based on the data from the TREND registry, ambulatory blood pressure monitoring in addition to office BP should be used for patient selection as well as for monitoring response to RDN. Furthermore, criteria for optimal patient selection are suggested.

## Background

With a prevalence of 5–30% in the hypertensive population [1], resistant hypertension (RH) is an important health problem and associated with high risk of cardiovascular events [2]. In light of the recently published SPRINT study [3], it is crucially important to have multiple drug treatment strategies and interventional procedures at disposal to treat as many patients as possible close to blood pressure (BP) targets. Since catheter-based endovascular sympathetic renal denervation (RDN) was introduced in 2008, it has become an additional treatment option [4]. The Symplicity HTN-1 and -2 studies [5, 6] proved the feasibility of the procedure and showed positive results with a low complication rate. They observed BP reductions of 20–30 mmHg.

Accordingly, RDN is a class IIb level C indication for treatment of RH by the 2013 ESC/ESH guidelines on the management of hypertension [1]. The guidelines propose careful patient selection and use in hands of experienced centres and operators. However, the Symplicity HTN-1 and -2 studies have been criticised for their non-standardized diagnostic pathway to confirm true RH and their controlled but non-blinded design [7, 8]. The Symplicity HTN-3 study [9] was the first blinded randomized sham-controlled trial of RDN. It confirmed safety but could not prove a significant effect of renal denervation on BP over sham procedure with a superiority margin of 5 mmHg. Indeed, the decline in office BP was not significantly different between both groups after 6 months (RDN group -14±24 mmHg vs. sham group -12±26 mmHg).

Since most of the studies dealing with RDN have a limited sample size and do not reflect a real-life scenario, their results do not transfer easily into clinical routine settings [10]. Moreover, the most commonly used surrogate of effective RDN treatment is the change in office blood pressure, despite its limited prognostic value in individual patients compared to home [11] or ambulatory blood pressure (ABP) [12, 13]. ABP monitoring (ABPM) has already become an essential part in the diagnostic pathway of hypertensive patients in national and international guidelines [14, 15] and is recommended for RDN studies [16]. However, only a minority of studies report ABPM data. In Symplicity HTN-1 and -2 trials, less than 45% of patients were evaluated by ABP monitoring at baseline and 6 months after procedure [17, 18]. It is well established that mean 24-h BP reductions and ABP responder rates (classified as mean 24-h SBP reduction ≥ 5 mmHg [19, 20] or ≥ 10 mmHg [21, 22]) are consistently less pronounced across all RDN trials as well as drug treatment trials, compared to office BP changes. To address these concerns, the Austrian Society of Hypertension created the Austrian Transcatheter RENal Denervation (TREND) Registry in 2011 with emphasis on ABPM to monitor safety and efficacy of all RDN procedures performed in Austria [23].

This is the first analysis of the data gathered by the Austrian TREND Registry, reporting efficacy and safety of RDN with respect to office and ambulatory BP in a real-life setting. The registry was built according to general applicable quality criteria for registries [10].

## Methods

### Study population

All participating centres were encouraged to select and evaluate patients for RDN according to the recommendations of the Austrian [24] and the European Society of Hypertension [25]. The Austrian Society of Hypertension suggested the enrolment of patients according to the diagnosis of uncontrolled hypertension (based on ABPM) and high cardiovascular risk. Restriction by the Austrian social security limit the reimbursement of RDN in Austria. Thus, only patients with long-lasting history of resistant hypertension underwent the procedure. Centres first ruled out secondary or treatable causes of hypertension by medical history, physical examination and routine laboratory investigations according to guidelines [1]. If necessary, additional diagnostic tests (e.g. ultrasonography, aldosterone-renin ratio, urinary cortisol and metanephrine secretion) were carried out. Secondly, they optimised medical and non-medical treatment including co-medications (with at least 5 antihypertensive medications at maximum tolerable doses). Thirdly, they evaluated adherence to therapy. Medication intake was confirmed by direct questioning. If necessary, actions to improve adherence were taken, e.g. the use of drug combinations in single pills and avoidance of drug interactions. We documented all types of antihypertensives and all changes of medication at time of inclusion and during follow-up. Thereafter, patients that retained a 24-h BP above 145/90 mmHg were eligible for the RDN procedure. Exclusion criteria were (1) a reduced kidney function (estimated glomerular filtration rate ≤ 45 mL/min) and (2) incompatible anatomy of the renal artery.

The Austrian guidelines recommended documenting all RDN procedures and related patient characteristics into the nationwide Austrian TREND Registry. International and national proctors assisted all operators for their first procedures to enhance quality of the procedures. Designated representatives of each centre confirmed completeness of patient-enrolment into the TREND Registry. A full list of participating centres is listed in the online appendix (S1 Table).

### Patient management

Office BP and ABP readings were conducted according to international guidelines [1]. Office BP was measured oscillometrically with validated automatic devices on the upper arm at two different occasions after 5 minutes’ rest. All centres used validated ABP monitors working oscillometrically. Recommended measurement intervals were 15 minutes during daytime and 30 minutes during night-time [26]. According to guidelines, 70% of ABP measurements should be valid. We did not document quality of ABP measurements in the registry. Patient management, choice of drug therapy, device selection for RDN as well as vascular access site remained at the discretion of each individual centre.

### The Austrian TREND Registry

The TREND Registry collected data via a web interface. Its frontend is located at the website of the Austrian Society of Hypertension (www.hochdruckliga.at). The Institutional Review Board of the Medical University Graz approved the registry (23–421 ex 10/11). Patient data collection was anonymous; respective centres could identify patients by their individual centre-based identification number only. The TREND Registry went online in April 2011 and all 20 Austrian centres performing RDN were invited to participate.

Baseline assessments comprised patients’ demographics, current antihypertensive treatment, comorbidities, office and ambulatory blood pressure. Additionally, presence of end-organ damage and kidney function (creatinine, estimated glomerular filtration rate, urinary albumin/creatinine ratio) were included. Following the documentation of procedural and safety details, follow-up (F/U) was recommended at 2–6 weeks, 3 and 6 months and on a yearly basis thereafter. Suggested F/U documentation included office BP, ABP, renal function, antihypertensive treatment and long-term safety. To ensure adherence to drug therapy, patients were encouraged to keep a diary.

### Subgroups

The Austrian Society of Hypertension suggested RDN for patients on multiple drug treatment, with a mean 24-h blood pressure > 145/90 mmHg [24]. This 24-h BP threshold has been chosen because its equivalent is an office BP of 160/100 mmHg [27]. However, this scientific statement was released after the introduction of RDN. Therefore, 40% of patients whose data were entered into the registry did not fulfil all of these criteria. To compare the characteristics and outcome of patients who satisfied these criteria with those who did not, we performed an additional analysis and formed two subgroups. Group A consisted of all patients with a mean baseline 24-h blood pressure > 145/90 mmHg. All remaining patients were summarized ingroup B.

### Data analysis

Patients’ data entered into the registry prior to December 31, 2014 were analysed. For univariate analysis, pairwise deletion was applied to missing data. All variables were reported as mean ± standard deviation, median (interquartile range) or count (proportion), where appropriate. BP values were always expressed as mean ± standard deviation to be comparable to other studies [5, 9, 17, 18, 28–32]; median values can be found in S2 Table. We used the Wilcoxon signed rank test for paired sample analysis. The Mann-Whitney U-Test and Fisher’s exact test were used for unpaired samples. We defined responders as follows: Office BP responders had to have a reduction of at least 10 mmHg of Office SBP after 6 months. [5, 6]. ABP responders had to have a 24-h ABP reduction of at least 5 mmHg after 6 months [19, 33]. Changes of systolic office and 24-h ambulatory BP between baseline and after 6 months were compared using the Pearson correlation coefficient. For logistic regression, ABP responder was defined as dependent variable. All baseline systolic and diastolic BP measurements (office, mean 24-h, mean day-time, mean night-time), age, gender, baseline creatinine level, estimated glomerular filtration rate, comorbidities, body mass index, number of antihypertensive medications, number of ablations and all significant predictors found in univariate analysis were considered as possible confounders. We included significant variables stepwise, using Wald’s test and excluded incomplete datasets from the regression analysis. The estimated glomerular filtration rate was calculated using CKD-EPI formula [34]. Unless stated otherwise, parameters were missing in < 5% of patients. A two-sided significance level of 0.05 was applied to all calculations. Data were analysed by IBM^®^ SPSS^®^ Statistics 20 (IBM Corporation, Armonk, NY). Graphs were designed with SigmaPlot^®^ 11.0 (Systat Software, San Jose, CA).

## Results

### Baseline characteristics

From March 2011 to December 2014, data from 407 consecutive patients at 14 centres were entered into the registry. Table 1 lists the baseline characteristics. Median age was 63 (54–69) years and 42% were female. Patients were on antihypertensive treatment for a median of 10 years (interquartile range 7–15; n = 128). Average office BP was 170±16/94±14 mmHg; average 24-h ABP was 151±18/89±14 mmHg (n = 359). In total, 98% of patients had a systolic office BP >140 mmHg and 91% a systolic 24-h ABP >130 mmHg, respectively. At baseline, patients received a median of four (interquartile range 4–5) different antihypertensive medications. Most prevalent comorbidities were coronary artery disease (37%), diabetes mellitus (36%) and cerebrovascular disease (12%).

**Table 1:** Main baseline characteristics of treated patients.

	All patients (n = 407)	Subgroup A (n = 245)	Subgroup B (n = 162)	P value
**Epidemiology**				
**Centres**	14	13	12	n/a
**Women**	171 (42)	102 (42)	69 (43)	0.918
**Age, years**	63 (54–69)	62 (52–68)	65 (56–71)	0.004*
**BMI, kg m**^**-2**^	30 (27–33)	30 (28–34)	29 (26–33)	0.007*
**Comorbidities**				
**Serum creatinine, μmol L**^**-1**^	84 (71–98) ^b^	84 (71–98) ^d^	84 (71–97) ^c^	0.709
**eGFR, ml min**^**-1**^ **per 1.73 m**^**2**^	78 (62–91) ^b^	77 (62–91) ^d^	78 (62–91) ^c^	0.094
**DM type 1**	1 (0.2)	1 (0.4)	0	0.600
**DM type 2**	133 (33)	83 (34)	50 (31)	0.518
**Coronary artery disease**	143 (37)	82 (34)	61 (41)	0.112
**Peripheral vascular disease**	14 (4)	7 (3)	7 (5)	0.406
**Cerebral vascular disease**	44 (12)	26 (11)	18 (12)	0.870
**Secondary arterial hypertension** ^**b**^				
**Obstructive sleep apnea**	17 (9)	14 (10)	3 (5)	0.405
**Hyperaldosteronism**	2 (0.5)	2 (0.8)	0	0.580
**Other**	0	0	0	n/a
**Medication**				
**No. of antihypertensive medications**	4 (4–5)	5 (4–6)	4 (3–5)	0.031*
**ACE inhibitors**	98 (26) ^f^	59 (25)	39 (26) ^f^	0.811
**Angiotensin receptor blockers**	199 (52) ^f^	119 (51)	80 (54) ^f^	0.528
**Alpha blockers**	123 (32) ^f^	79 (34)	44 (30) ^f^	0.433
**Beta blockers**	312 (82) ^f^	197 (84)	115 (78) ^f^	0.172
**Calcium antagonists**	253 (66) ^f^	163 (70) ^f^	90 (61) ^f^	0.075
**Any diuretic**	324 (85) ^f^	198 (85)	126 (85) ^f^	1.000
**Loop diuretics**	27 (14) ^a^	21 (15) ^b^	6 (10) ^a^	0.497
**Hydrochlorothiazide**	157 (80) ^a^	111 (80) ^b^	83 (56) ^a^	1.000
**Aldosterone antagonist**	84 (22) ^e^	57 (25)	27 (18) ^f^	0.165
**Minoxidil**	23 (12) ^a^	16 (12) ^b^	7 (12) ^a^	1.000
**Renin inhibitors**	95 (25) ^f^	64 (28)	31 (21) ^f^	0.181
**Central antisympatholytics**	124 (33) ^f^	81 (35)	43 (29) ^a^	0.218
**Blood pressure, systolic/diastolic, mmHg**				
**Office BP**	171±18 / 94±15 ^d^	173±18 / 96±16 ^d^	166±17 / 90±12 ^c^	0.002* / 0.001*
**24-h mean BP**	151±18 / 89±14 ^e^	159±15 / 95±13	132±9 / 77±7 ^d^	<0.001*/ <0.001*
**Daytime mean BP**	152±18 / 90±14 ^e^	161±15 / 96±13 ^f^	134±9 / 79±8 ^c^	<0.001*/ <0.001*
**Nighttime mean BP**	142±21 / 82±15 ^e^	151±19 / 87±14 ^f^	126±14 / 72±10 ^c^	<0.001*/ <0.001*
**Blood pressure behaviour**				
**Reverse dipper**	71 (21) ^e^	45 (20) ^f^	26 (23) ^c^	0.482
**Mild dipper**	160 (47) ^e^	113 (50) ^f^	47 (42) ^c^	0.167
**Regular or extreme dipper**	107 (32) ^e^	68 (30) ^f^	39 (35) ^c^	0.387
**Isolated hypertension**	123 (39) ^d^	74 (36) ^e^	49 (45) ^c^	0.080
**Pseudohypertension**	18 (7) ^e^	0 (0) ^d^	18 (23) ^a^	<0.001*
**Masked hypertension**	5 (2) ^c^	1 (0.5) ^d^	4 (5) ^a^	0.027*

ACE, angiotensin converting enzyme; BMI, body mass index; eGFR, estimated glomerular filtration rate; DM, diabetes mellitus. Data available in > 95% of cases, except ^a^ < 50%, ^b^ 50–59%, ^c^ 60–69%, ^d^ 70–79%, ^e^ 80–89%, ^f^ 90–95%.

* p < 0.05

### Subgroups

A subgroup of 245 patients (60%) met the criteria of mean 24-h BP above 145/90 mmHg (group A). These patients were significantly younger, had a higher body mass index, and received more antihypertensive medications than group B (Table 1). Mean 24-h BP in group A was 159/95 mmHg compared to 132/77 mmHg in group B. This difference was significant (p < 0.001). Furthermore, the average office BP in group A was significantly higher, but the difference was much smaller (173/96 mmHg vs. 166/90 mmHg). There were no significant differences in comorbidities.

### Procedural details and safety

Procedural details were available for 279 patients (69%). Antihypertensive therapy was paused during the procedure in 44% of cases. Most of the patients were treated with Symplicity^™^ Renal Denervation Systems (Medtronic Inc., Minneapolis, MN; n = 380, 95%). Due to the very small number of patients treated with Symplicity Spyral^™^ Renal Denervation Systems (Medtronic Inc., Minneapolis, MN; n = 11, 3%) and EnligHTN^™^ system (St. Jude Medical Inc., St. Paul, MN, n = 8, 2%), there is no comparison of these different systems concerning adverse events in our study. In subgroups A and B, a median sum of 11 (9–12) and 10 (9–12) points in both renal arteries were ablated (p = 0.412, S3 Table). Periprocedural complication rate was 2.5% (n = 7) with no significant difference between the subgroups (p = 0.712).

The following complications were documented: inguinal haematoma requiring intervention (n = 1), renal arterial dissection requiring stenting (n = 1), pseudo-aneurysm of the femoral artery (n = 2), dissection of the abdominal aorta (treated conservatively, n = 1), spasm of the renal artery (n = 1) and therapy-resistant hypotension (n = 1). All complications were managed successfully in the catheter room. Periprocedural mortality was 0%. Two patients required percutaneous transluminal renal angioplasty for renal artery stenosis 72 and 452 days after the intervention.

### Follow-up and BP changes

**Fig 1:**
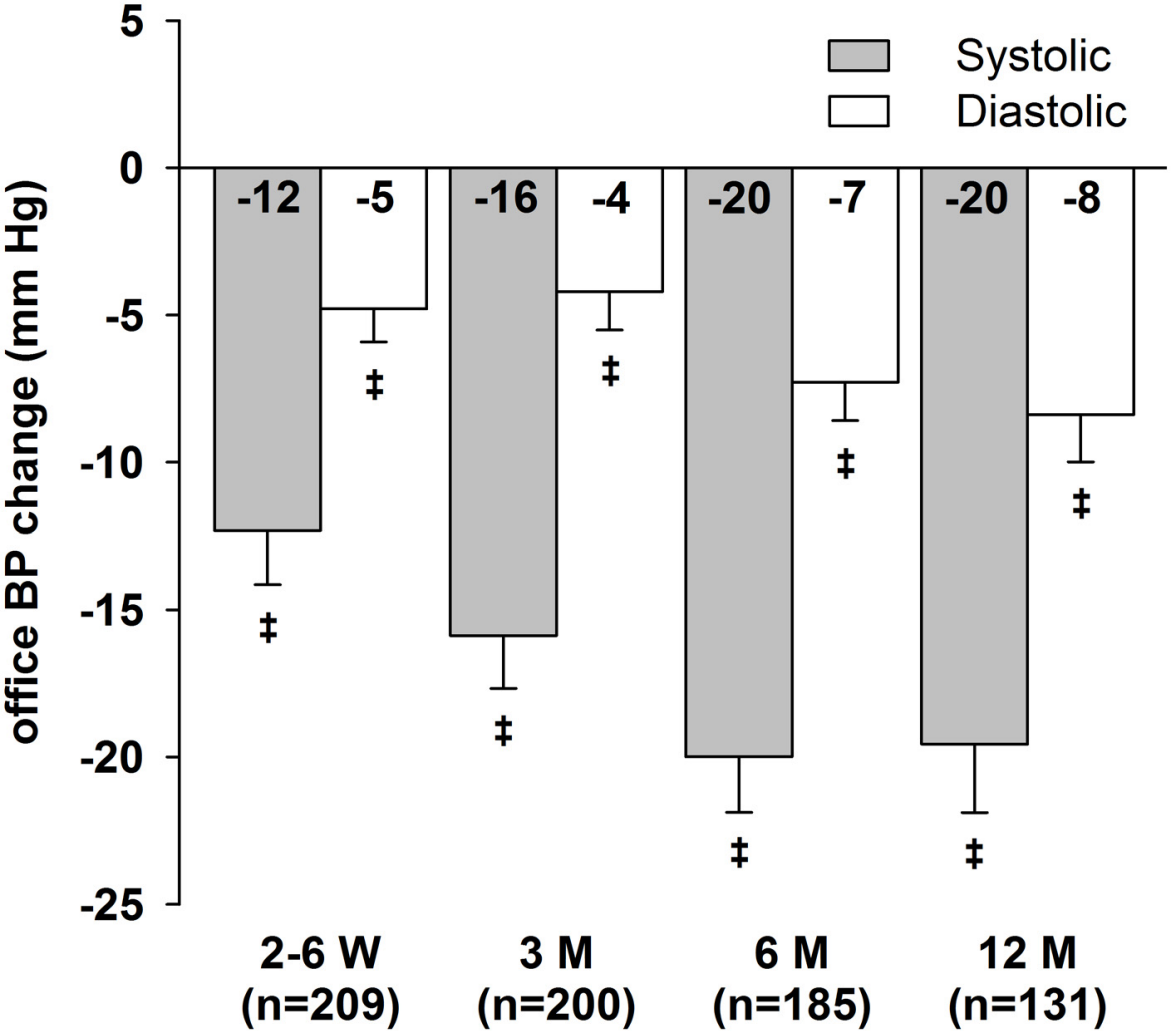
Mean office BP changes after RDN over 12 months of follow-up. Error bars represent standard error of means. ^‡^ p<0.001.

**Fig 2:**
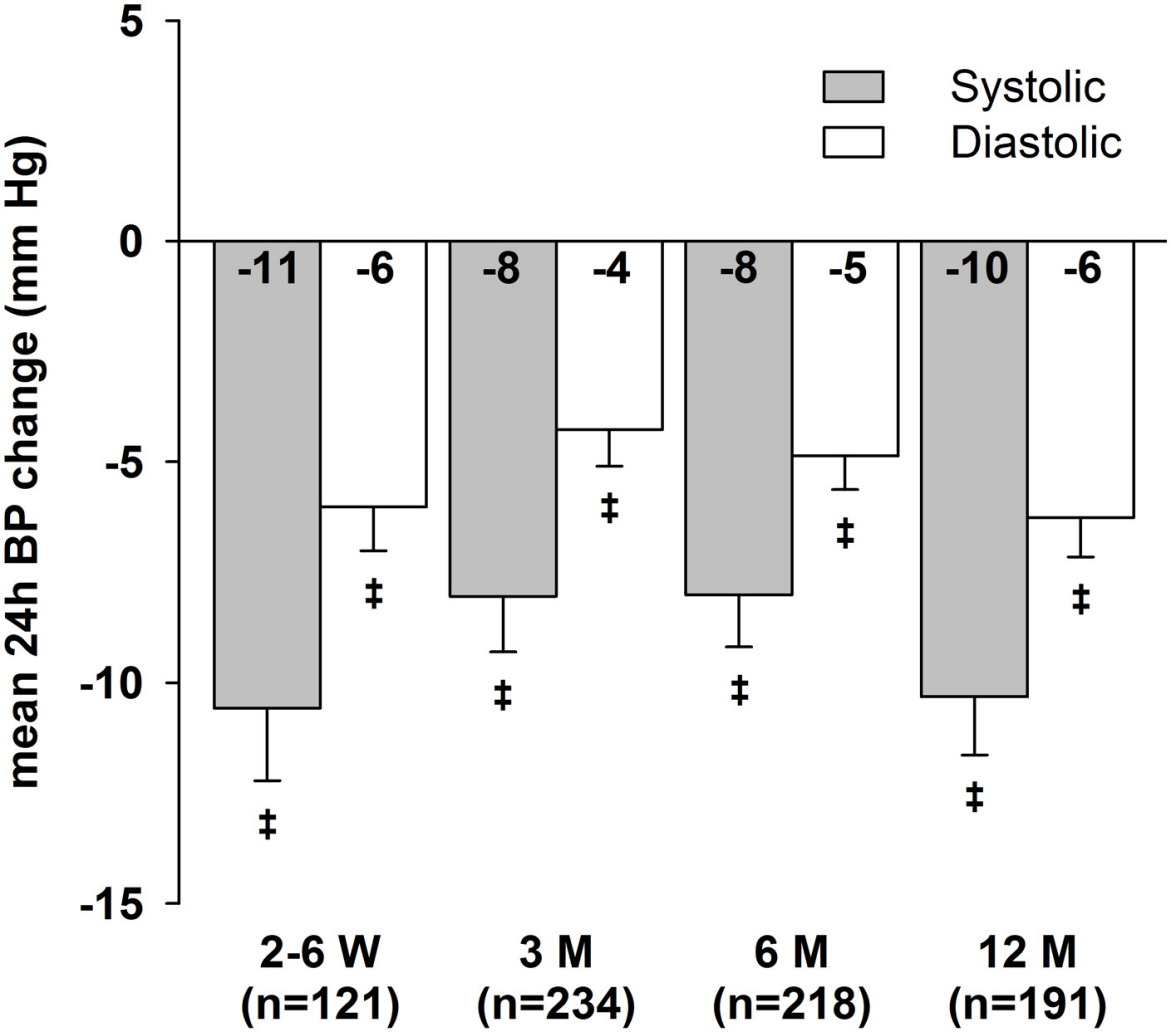
Mean 24-h BP changes after RDN over 12 months of follow-up. Error bars represent standard error of means. ^‡^ p<0.001.

Median follow-up time was 1 year (205–383 days). Post-procedural ABP data were available for 319 patients (78%). Figs 1 and 2 illustrate office and mean 24-h BP changes compared to baseline. All office BP and ABP reductions were significant compared to baseline (Table 2). In the course of the follow-up, we observed a slight decrease in renal function (-2 ml/min/1.73m^2^ after 1 year). The number of different antihypertensive drugs used decreased after RDN; this reduction was significant after 3 and 12 months. Office BP (systolic/diastolic) dropped by 20.0±25.7/7.3±17.7 mmHg after 6 months, respectively. Mean 24-h BP decreased by 8.0±17.5/4.9±11.3 mmHg 6 months after the procedure. The office BP responder rate after 6 months as defined by the Symplicity studies [5, 6], was 69% (128 of 185 patients). We observed a 24-h BP reduction ≥ 5 mmHg in 120 of 220 patients (55%) and 44% (67 of 154 patients) were both office BP and ABP responders. In total, 30% of patients achieved the systolic office BP goal of ≤ 140 mmHg at every follow-up and 22% of patients achieved the systolic 24-h BP goal of ≤ 130 mmHg, respectively.

**Table 2:** Responses to RDN at 2–6 weeks, 3, 6, and 12 months after procedure.

	2–6 weeks		3 months		6 months		12 months	
Blood pressure (mmHg)	systolic	diastolic	systolic	diastolic	systolic	diastolic	systolic	diastolic
**Office BP, n**	(n = 212)		(n = 206)		(n = 188)		(n = 134)	
**Absolute**	158±24	89±13	153±22	89±13	151±22	87±15	153±23	88±16
**Change to baseline**	-12±27 ^b^	-5±16 ^b^	-16±25 ^b^	-4±18 ^b^	-20±26 ^b^	-7±18 ^b^	-20±27 ^b^	-8±18 ^b^
**Mean 24-h BP, n**	(n = 130)		(n = 253)		(n = 239)		(n = 208)	
**Absolute**	142±15	84±11	140±18	83±13	139±16	83±12	137±17	82±13
**Change to baseline**	-11±18 ^b^	-6±11 ^b^	-8±19 ^b^	-4±13 ^b^	-8±17 ^b^	-5±11 ^b^	-10±18 ^b^	-6±12 ^b^
**Mean daytime BP, n**	(n = 111)		(n = 241)		(n = 225)		(n = 198)	
**Absolute**	144±15	87±11	141±18	85±14	141±16	85±13	139±18	84±13
**Change to baseline**	-10±19 ^b^	-4±11 ^b^	-8±20 ^b^	-4±12 ^b^	-7±18 ^b^	-4±10 ^b^	-10±19 ^b^	-5±12 ^b^
**Mean nighttime BP, n**	(n = 110)		(n = 237)		(n = 221)		(n = 192)	
**Absolute**	137±17	79±14	132±19	77±13	133±19	77±13	131±19	76±13
**Change to baseline**	-10±18 ^b^	-5±12 ^b^	-8±21 ^b^	-4±13 ^b^	-7±21 ^b^	-4±12 ^b^	-9±21 ^b^	-5±12 ^b^
**Medication**								
**Number of antihypertensive medications**	(n = 136)		(n = 142)		(n = 134)		(n = 267)	
**Absolute**	5 (4–6)		4 (4–5)		5 (4–6)		4 (3–5)	
**Change to baseline**	0 (0–0)		0 (-1–0) ^a^		0 (0–0)		0 (-1–0) ^a^	
**Renal function**								
**eGFR, ml min**^**-1**^ **per 1.73m**^**2**^	(n = 174)		(n = 182)		(n = 127)		(n = 112)	
**Absolute**	80 (64–93)		75 (62–90)		74 (63–86)		74 (59–84)	
**Change to baseline**	-0.5 (-7–5)		-0.7 (-9–4) ^a^		-2 (-11–7)		-2 (-11–5) ^a^	

All values are presented as mean±SD. BP, blood pressure; eGFR, estimated glomerular filtration rate.

^a^ p<0.05;

^b^ p<0.001

here were no significant differences between patients treated with different devices in the 24-h and office BP responders (based on BP changes after 6 months). However, ambulatory daytime and night-time SBP changes were more pronounced in Symplicity Spyral group after 1 month (p≤0.001 for both). There was no follow-up ABPM data available for the EngligHTN group.

### Correlation of office and 24-h BP changes

**Fig 3:**
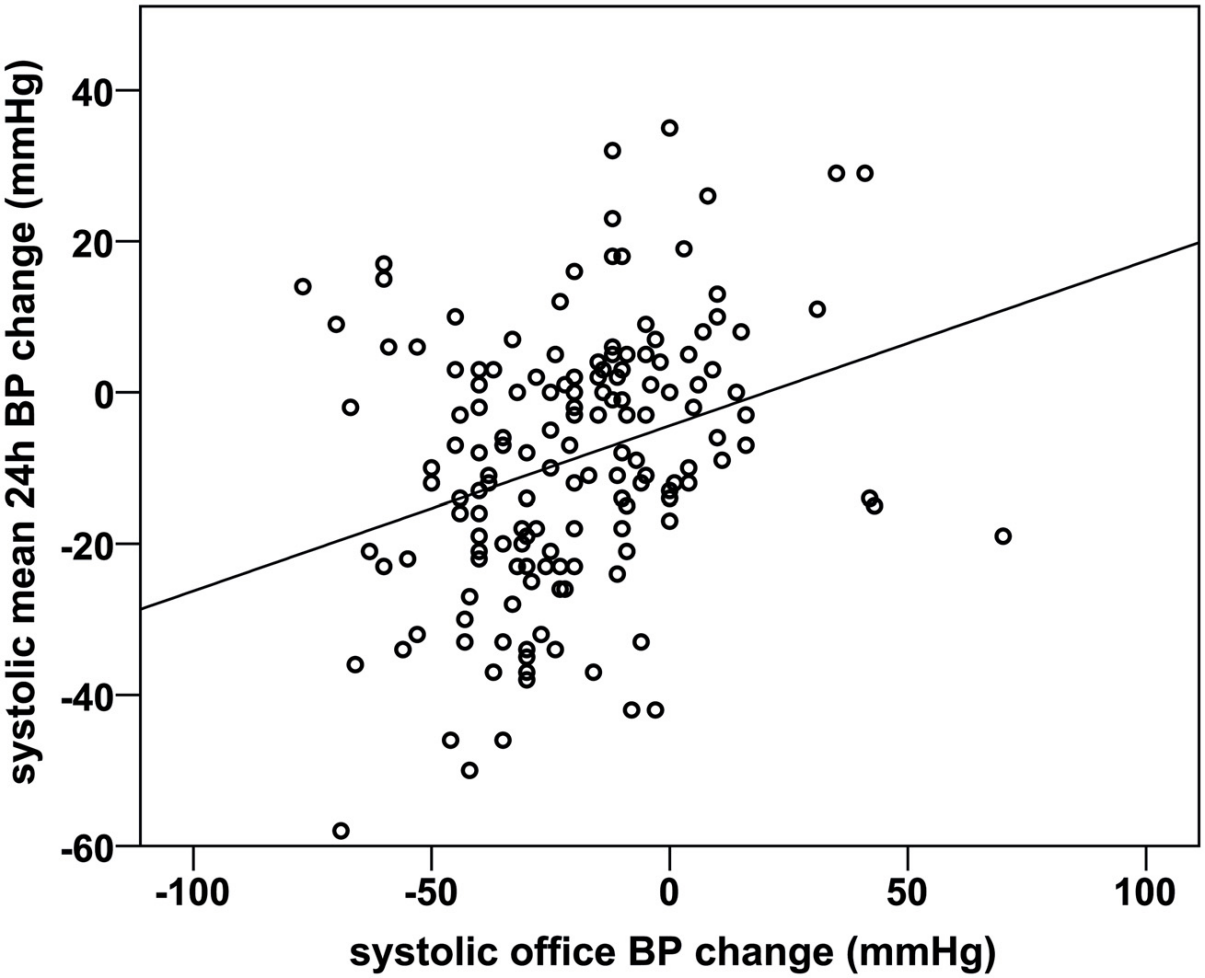
Correlation between systolic mean 24-h and office BP changes at baseline and 6 months after procedure (n = 154). Pearson correlation 0.303 (p<0.001).

The correlation of systolic office and mean 24-h BP changes 6 months after procedure (n = 154) is demonstrated in Fig 3. The Pearson coefficient was 0.303 (p<0.001). Office BP responder predicted ABP responders with a sensitivity of 77% and a specificity of 37% (p = 0.073, S4 Table).

### Predictors of ABP responder

High systolic and diastolic 24-h, day-time and night-time ABP, as well as pulse pressures, were predictors of clinically relevant change of systolic 24-h BP (BP reduction ≥ 5 mmHg; n = 220). In logistic regression analysis, a high mean 24-h SBP, a low office systolic BP and a low mean night-time diastolic BP predicted a relevant 24-h SBP reduction (n = 156, Table 3). The resulting model could predict ABP responders with a sensitivity of 81% and a specificity of 74%.

**Table 3:** Predictors of 24-h mean systolic blood pressure reduction ≥ 5 mmHg after 6 months.

Parameter	OR	95% CI	p
**Mean 24-h systolic BP, per 10 mmHg**	3.261	2.175 to 4.888	<0.001
**Office systolic BP, per 10 mmHg**	0.676	0.515 to 0.888	0.005
**Mean nighttime diastolic BP, per 10 mmHg**	0.626	0.429 to 0.913	0.015

BP indicates blood pressure; OR, odds ratio; CI, confidence interval.

### Subgroups

In group A, 24-h BP reductions after the procedure were significantly more apparent compared to group B (p < 0.01 at every follow-up). Furthermore, with a mean 24-h BP change of -13.7±16.8 / -8.2±11.6 mmHg after 6 months (n = 137), ABP responder rate was significantly higher (group A 70% vs. group B 29%, p<0.001). Office responder rate did not differ between subgroups (68% vs. 69%, p = 0.621).

## Discussion

The analysis of the investigator-initiated Austrian TREND registry revealed three major findings: First, we observed a significant and sustained BP lowering effect reflected in both office BP readings and ABP monitoring. This effect was strongest in patients with a mean baseline 24-h BP ≥ 145/90 mmHg. Second, renal denervation was a safe procedure with a low complication rate. Third, ABP responders (decrease of ≥ 5 mmHg ambulatory SBP after RDN) were not superimposable to office BP responders (decrease of ≥ 10 mmHg of office SBP after RDN).

### Efficacy

The Symplicity HTN-3 study [9] raised concerns about the efficacy of RDN [35]. However, according to the data from the Austrian TREND registry, a considerable proportion of patients benefitted from the procedure. ABP and office BP responder rate in this study were 55% and 69%, respectively. The mean 24-h BP reduction in this analysis of -8.0/-4.9 mmHg after 6 months is comparable to other studies [19, 20, 22]. The results derived from the Austrian TREND registry support the findings of the report on the Global Symplicity Registry (GSR), which included 998 patients treated in 134 centres [29]. We found even more pronounced office BP reductions (-20 mmHg) than the GSR report which reported a reduction of -12 mmHg in office SBP after a follow-up of 6 months. This might be due to a higher level of baseline office BP in our cohort. The ABP reductions in our study are similar to the GSR (-7 mmHg).

There are important differences between the GSR and the Austrian TREND Registry. The Austrian Registry was investigator-initiated and had a longer median follow-up period of one year compared to 6 months in the GSR. Additionally, ABP was reported in a higher proportion of patients (88%) compared to 51% in the GSR.

The Hawthorne effect has been discussed as potential explanation for the encouraging results of open RDN trials but failed blinded Symplicity HTN-3 study [8, 35, 36]. However, a recent study found no increase in therapy adherence in patients treated with RDN [37]. In our study, there was even a slight decrease in prescribed antihypertensive medications, which was significant after 3 and 12 months. Whereas RDN is not considered as replacement for optimal medical treatment [38], the antihypertensive effect seems to be the largest where both medical and interventional treatment options were used in combination [39].

When comparing patients treated with different RDN devices, we found a significantly higher baseline 24-h SBP in patients with Symplicity Spyral, and additionally a more pronounced decrease of daytime and nighttime SBP after 1 month. There were no significant differences of responder rate after 6 months. As a limitation, the sample sizes of patients treated with Symplicity Spyral or EnligHTN were too low to reliably confirm or dismiss differences between different types of devices.

### Safety

Most studies dealing with RDN report a periprocedural complication rate below 5% [5, 9, 22, 29, 30]. The majority of events were not related directly to the denervation itself, but to the vascular access site (e.g. hematoma, pseudo-aneurysm of the femoral artery). Data from the Austrian TREND registry confirm this previous findings, with a periprocedural complication rate of 2.5%. RDN can be considered a safe interventional procedure.

Renal artery stenosis is a feared long-time complication after RDN [40]. However, only two cases (0.5%) of renal artery stenosis were reported in the Austrian TREND registry and similar incidences in other studies [9, 17, 18, 22, 41, 42]. Kidney function also remained stable in our study. The decline of estimated glomerular filtration rate of 2 ml/min/1.73m² after 12 months possibly reflects a normal decline caused by the aging of patients. Symplicity HTN-3 study showed no significant difference in long-term adverse events compared to sham control [9].

### ABP vs. office BP

Our study found a weak, but statistically significant correlation between systolic office BP and ABP changes after procedure. When analysing BP responders to RDN individually, ABP responders and office BP responders were not congruent. Only 61% of office BP responders were also ABP responders. On the contrary, 23 percent of ABP responders were not classified as office BP responders. These results support the well-known fact that in individual patients office BP is not representative for ambulatory BP [11, 13].

To our knowledge, there is no other study that scrutinises the differences of office BP and ABPM in patients undergoing RDN therapy in detail. Many studies claimed that the incidence of ABP responders was lower than those of office BP responders [22, 29], but there were no details about the association between those two groups reported. Our data strongly suggest ABPM to be of crucial importance not only for patient selection prior to RDN, but also for monitoring of efficacy after the procedure.

As shown in other studies [43, 44], persisting white coat effects or a large variance of office BP readings may explain this effect. As a result of these findings, we advise to interpret single office BP measurements with caution in patients after RDN.

### Predictors of ABP responders

Our data support the hypothesis that 24-h baseline SBP is the best predictor for the efficacy of the treatment [21, 31, 45]. The GSR reported a subgroup of severely hypertensive patients (office SBP ≥ 160 mmHg, ambulatory SBP ≥ 135 mmHg and ≥ 3 antihypertensive drugs), in whom ambulatory SBP dropped by 9 mmHg. This group was comparable to subgroup A in this analysis, defined as either systolic and/or diastolic ABP ≥ 145 / 90 mmHg despite antihypertensive combination therapy. Both, the subgroup A of our registry and the severe hypertension group of the GSR, experienced the highest drop of BP after the procedure.

Besides high systolic 24-h BP, other studies also suggested preserved renal function, a high number of ablation attempts, cardiovascular comorbidities and low patient age (<65 years) as potential predictors [19, 21, 22, 31, 45]. We could not replicate any of these parameters as confounders in univariate or multivariate analysis.

Recent studies claimed that BP reductions would be weaker in patients with isolated systolic hypertension [46] or high central pulse pressure [47]. When we controlled for 24-h mean SBP, low office SBP and low mean nighttime diastolic BP were predictive of clinically significant ABP reduction after 6 months. This suggests that patients with masked hypertension are especially prone to respond to RDN. Consequently, it goes in line with the GSR that proved effectiveness in a patient subgroup of masked hypertension [29].

### Limitations

The strength of this study is limited by the fact that not all patient data were entered, as the participation in the registry was encouraged but not mandatory. While 6-months ABPM data availability of 59% was superior to former studies [29], data still has to be interpreted with some caution as selection bias might have occurred.

While we sought to exclude effects other than the ablation procedure that could cause a reduction of BP levels over time, the analysis remains subject to certain limitations in this regard. Since regression to the mean phenomenon and the regression of the white coat effect may lower BP readings at subsequent follow-up visits, our data might over-estimate especially office BP reductions.

We measured adherence to antihypertensive therapy similar to the GSR investigators. Drug prescriptions and changes of medications were documented in the registry. However, urine analysis or pill count for proving accurate drug intake was not available.

### Conclusion

The Austrian TREND Registry demonstrated efficacy and safety of RDN in patients with a history of long-lasting hypertension. The results underline the importance of ambulatory blood pressure for defining suitability of patents and for documentation of an effective BP response to RDN. Patients with a baseline 24-h BP above 145/90 mmHg benefitted most from the procedure.

## Supporting Information

S1 Dataset All data (CSV format). (CSV)

S2 Dataset All data (SAV format). (SAV)

S1 Table Participating centres. (PDF)

S2 Table Baseline BP and responses to RDN at 2–6 weeks, 3, 6, and 12 months after procedure, expressed as median (interquartile range). p<0.001 for all BP changes.
(PDF)

S3 Table Anatomical and procedural details. (PDF)

S4 Table Office BP and 24-h BP responders among patients with available data (n = 154). (PDF)
