# Supplementary material for: Effects of Renal Denervation Documented in the Austrian National Multicentre Renal Denervation Registry
Source: PLoS One. 2016 Aug 16;11(8):e0161250. doi: 10.1371/journal.pone.0161250 (PMC4987037; doi:10.1371/journal.pone.0161250)
Supplement: S1 Table — (PDF) [file pone.0161250.s003.pdf]

Division of Cardiology, Department of Internal Medicine, Medizinische Universität Graz, Auenbruggerplatz 15, 8036 Graz, Austria
Department of Internal Medicine I, AKH Linz, Krankenhausstraße 9, 4020 Linz, Austria
Department of Internal Medicine II, Klinikum Wels-Grieskirchen, Grieskirchner Straße 42, 4600 Wels, Austria
Department of Internal Medicine III, Medizinische Universität Innsbruck, Anichstraße 35, 6020 Innsbruck, Austria
Department of Internal Medicine IV, Medizinische Universität Innsbruck, Anichstraße 35, 6020 Innsbruck, Austria
Division of Nephrology, Department of Internal Medicine III, Medical University Vienna, Waehringer Guertel 18-20, 1090 Vienna Austria
Department of Internal Medicine II, Krankenhaus der Elisabethinen Linz, Fadingerstraße 1, 4020 Linz, Austria
Department of Internal Medicine, Landeskrankenhaus Waidhofen an der Ybbs, Ybbsitzerstraße 112, 3340 Waidhofen/Ybbs, Austria
Third Department of Internal Medicine, Wilhelminenspital, Montleartstraße 37, 1160 Vienna, Austria
Department of Internal Medicine II, Paracelsus Medizinische Privatuniversität Salzburg, Müllner Hauptstraße 48, 5020 Salzburg, Austria
Department of Internal Medicine I, Krankenhaus St. Josef Braunau, Ringstraße 60, 5280 Braunau, Austria
Fifth Medical Department, Kaiser-Franz-Josef-Spital, Kundratstraße 3, 1100 Vienna, Austria
Privatklinik Mariahilf, Radetzkystraße 35, 9020 Klagenfurt, Austria
Department of Internal Medicine, Krankenhaus St. Vinzenz, Sanatoriumstraße 43, 6511 Zams, Austria

Department of Internal Medicine II, Krankenhaus Wiener Neustadt, Corvinusring 3-5, 2700

Wiener Neustadt, Austria
