# Supplementary material for: Effects of Renal Denervation Documented in the Austrian National Multicentre Renal Denervation Registry
Source: PLoS One. 2016 Aug 16;11(8):e0161250. doi: 10.1371/journal.pone.0161250 (PMC4987037; doi:10.1371/journal.pone.0161250)
Supplement: S2 Table — p<0.001 for all BP changes. (PDF) [file pone.0161250.s004.pdf]

	baseline		2-6 weeks		3 months		6 months		12 months	
**Blood pressure (mmHg)**	**systolic**	**diastolic**	**systolic**	**diastolic**	**systolic**	**diastolic**	**systolic**	**diastolic**	**systolic**	**diastolic**
**Office BP, n**	(n=297)		(n=212)		(n=206)		(n=188)		(n=134)	
**absolute**	170 (160-180)	90 (84-100)	156 (141-171)	90 (80-95)	150 (140-167)	87 (80-95)	150 (135-161)	85 (80-95)	148 (135-167)	86 (87-96)
**change to baseline**			-15 (-29 – 5)	-5 (-15 – 5)	-17 (-32 – -2)	-5 (-15 – 5)	-20 (-37 – -5)	-6 (-19 – 4)	-21 (-37 – 0)	-9 (-9 – -20)
**Mean 24-h BP, n**	(n=359)		(n=130)		(n=253)		(n=239)		(n=208)	
**absolute**	149 (137-164)	88 (79-98)	142 (133-153)	83 (75-91)	138 (126-151)	82 (73-92)	138 (128-149)	83 (74-90)	136 (125-146)	80 (74-87)
**change to baseline**			-9 (-22 – 3)	-5 (-13 – 1)	-9 (-21 – 5)	-4 (-11 – 3)	-8 (-20 – 3)	-5 (-13 – 2)	-9 (-23 – 3)	-4 (-13 – 2)
**Mean daytime BP, n**	(n=345)		(n=111)		(n=241)		(n=225)		(n=198)	

**absolute**	151 (138-165)	89 (80-100)	145 (133-155)	85 (78-96)	140 (127-152)	85 (75-94)	140 (129-150)	84 (75-93)	138 (126-148)	83 (75-90)
**change to baseline**			-8 (-23 – 6)	-3 (-11 – 4)	-9 (-21 – 3)	-4 (-11 – 4)	-8 (-20-4)	-4 (-10 – 2)	-10 (-24 – 5)	-4 (-12 – 3)
**Mean nighttime BP, n**	(n=338)		(n=110)		(n=237)		(n=221)		(n=192)	
**absolute**	141 (129-154)	80 (72-91)	136 (124-147)	76 (70-89)	132 (119-146)	76 (67-85)	132 (120-144)	76 (68-85)	127 (118-142)	75 (67-83)
**change to baseline**			-3 (-13 – 2)	-10 (-20 – 5)	-3 (-11 – 2)	-15 (-21 – 0)	-4 (-12 – 5)	-12 (-25 – -2)	-5 (-11 – 2)	-12 (-25 – 0)
