# Supplementary material for: Effects of Renal Denervation Documented in the Austrian National Multicentre Renal Denervation Registry
Source: PLoS One. 2016 Aug 16;11(8):e0161250. doi: 10.1371/journal.pone.0161250 (PMC4987037; doi:10.1371/journal.pone.0161250)
Supplement: S3 Table — (PDF) [file pone.0161250.s005.pdf]

	Subgroup A	Subgroup B	p value
**Anatomy**			
**additional left renal artery**	7% (10/147)	5% (3/65)	0.758
**additional right renal artery**	10% (15/147)	3% (2/66)	0.100
**Procedure**			
**total no. of ablations**	11 (9-12) (n=186)	10 (9-12) (n=93)	0.412
**total no. of 120s ablations**	10 (8-12) (n=110)	10 (7-11) (n=35)	0.087
**ablation of additional artery**	9% (2/23)	0% (0/5)	1.000
**total no. of ablations additional artery**	5.5 (2-9)	N/A	N/A
**contrast dye, ml**	95 (64-145) (n=87)	130 (90-200) (n=71)	0.073
**Complications**			
**renal arterial dissection**	0.0% (0/186)	1.1% (1/93)	0.355
**pseudoaneurysm**	1.0% (2/186)	0.0% (0/93)	0.548
**haematoma**	0.0% (0/186)	1.1% (1/93)	0.341
**hypotension**	0.5% (1/186)	0.0% (0/93)	1.000
**dissection of abdominal aorta**	0.5% (1/186)	0.0% (0/93)	1.000
**renal artery spasm**	0.5% (1/186)	0.0% (0/93)	1.000
**any complication**	2.7% (5/186)	2.2% (2/93)	0.712
