# Supplementary material for: Effects of Renal Denervation Documented in the Austrian National Multicentre Renal Denervation Registry
Source: PLoS One. 2016 Aug 16;11(8):e0161250. doi: 10.1371/journal.pone.0161250 (PMC4987037; doi:10.1371/journal.pone.0161250)
Supplement: S4 Table — (PDF) [file pone.0161250.s006.pdf]

		Office BP responder	No Office BP responder	p value
**Subgroup A**	**24-h BP responder**	53	17	0.012
	**No 24-h BP responder**	15	15	
**Subgroup B**	**24-h BP responder**	14	3	0.350
	**No 24-h BP responder**	27	10	
